# Time‐Varying Hormonal Treatment and Metastasis‐Free Survival Among ER+ Breast Cancer Patients: A Natural History Modelling Approach

**DOI:** 10.1002/sim.70504

**Published:** 2026-04-02

**Authors:** Letizia Orsini, Alessandro Gasparini, Kamila Czene, Keith Humphreys

**Affiliations:** ^1^ Department of Medical Epidemiology and Biostatistics Karolinska Institutet Stockholm Sweden; ^2^ Swedish e‐Science Research Centre Karolinska Institutet Stockholm Sweden; ^3^ Red Door Analytics AB Stockholm Sweden

**Keywords:** breast cancer screening, breast cancer survival, hormonal treatment effect, metastatic process, natural history model, time‐varying treatment

## Abstract

Breast cancer treatment depends on tumour subtypes. In particular, patients with oestrogen receptor‐positive (ER+) tumours are treated with hormonal therapy. In Sweden, the recommended treatment duration is five years, with current guidelines advising an additional five years for women at high risk of disease recurrence. However, the impact of extended therapy on metastatic progression has not been thoroughly quantified at the population level. In this article, we use a modelling approach to estimate the time‐varying effect of hormonal treatment on time to metastasis. The model is then used to compare 5‐ and 10‐year treatment durations at different tumour sizes. Rather than using a common statistical modelling approach, we incorporate the effect of endocrine therapy within a biologically inspired natural history model of breast cancer to accommodate key features of the expected treatment‐outcome relationship. We fitted the model using maximum likelihood and data from a cohort of 9,716 incident cases diagnosed with invasive ER+ breast cancer between 2005 and 2020. Based on our main model estimates, the 10‐year metastasis‐free survival would improve from 92.8% to 96.1% for a symptomatic patient with a 20 mm tumour with ten years (instead of five) of hormonal treatment. Our natural history model quantifies the impact of prolonged hormonal treatment on metastatic events in ER+ breast cancer patients, including features that are not captured by traditional statistical approaches. Results suggest a significant reduction in metastatic tumour growth rates during treatment, supporting the extension of endocrine therapy to 10 years for people with large tumours.

## 1 Introduction

Breast cancer is a very diverse disease and its treatments depend on the tumour subtype. For patients presenting tumours that are oestrogen receptor‐positive (ER+), defined in Sweden as at least 10% cells staining positive, the standard of care in Sweden has been 5 years of hormonal treatment, either tamoxifen or aromatase inhibitor, with the aim of avoiding a short recurrence of the disease. However, randomised clinical trials [1, 2, 3] have suggested that extending endocrine therapy beyond five years reduces late recurrences and improves survival outcomes. Since the mid‐2010s, Sweden has progressively adopted longer durations of endocrine therapy, extending standard treatment to 10 years [4], particularly for patients with a high risk of recurrence. Despite this shift, many patients discontinue therapy prematurely due to side effects.

Tamoxifen is a selective oestrogen receptor modulator (SERM) that works as an antagonist—it binds to the oestrogen receptors (ERs) on the breast cell to block other molecules from activating the receptor. It can therefore stop or slow the growth of oestrogen receptor‐positive metastatic tumours. Aromatase inhibitors instead work by inhibiting oestrogen production, which consequently can also limit the growth of these tumours [5].

Due to its side effects, hormonal treatment has a low compliance that has been estimated to be around 60%–82% in 3 years and 46%–73% in 5 years in different retrospective studies [6, 7, 8, 9, 10]. It is of clear clinical interest to quantify the effect of hormonal treatment on patients at a population level. Zeng et al. [11] evaluated the effect of extending hormonal treatment beyond 5 years on disease‐free survival (and overall survival) and reported a statistically significant hazard ratio (HR) of 0.72 [95% CI 0.55–0.95] for extending treatment after 5 years compared to stopping treatment after the first 5 years. In evaluating this HR, the authors implicitly assumed that treatment continued throughout the entire follow‐up period for all patients that had treatment extended beyond five years, and interpreted treatment as having an instantaneous effect on the hazard rate. They did that because, although they had information on prescription collection dates, it was not straightforward/possible to incorporate time‐varying effects in a relevant way using standard methods. In that study, follow‐up began 5 years after diagnosis.

Statistical approaches that could be used in this context, which model the association between time‐varying treatments and time to event outcomes, include Cox proportional hazard models for time dependent covariates [12], flexible parametric survival analysis [13], joint models of longitudinal markers and time to event outcomes [14], and, for causal inference, marginal structural models (MSMs) estimated with inverse probability of treatment weighting (IPTW) [15]. We have chosen to follow a rather different strategy, using biological motivations to capture what we believe are key features of the relationship between treatment and outcome in our setting. We return to the comparison with the more common statistical approaches in the Discussion.

The project described here aims to estimate the effect of hormonal treatment by incorporating the time under treatment, for each patient, into a statistical model of the natural history of breast cancer. We quantify the hormonal treatment effect by using a biologically inspired statistical model for the natural history of breast cancer.

Although the proposed model inevitably represents a vast simplification of the tumour progression process and the effect of treatment on patient's outcome (all statistical models are of course wrong, but latent variable models are particularly reliant on assumptions that are not easy to examine), the model has the advantage that it incorporates timing of treatment in a way that is biologically motivated. In contrast, a standard approach that models the hazard of diagnosis as a function of current treatment is certainly guilty of mishandling the time relationships between treatment and outcome. The aim here is to propose a framework that could help quantify the effect of treatment changes on patient outcomes, even though it is based on strong assumptions.

The paper is structured as follows. First, we describe the natural history model that we have developed, including the underlying assumptions about tumour growth, the metastatic seeding process, and how we incorporate the effects of endocrine therapy on metastasis‐free survival. Second, we derive the mathematical formulae that describe the likelihood for both the population that participates in the screening program and the population that does not. Third, we provide details about the Swedish cohort used to fit the model, which is the same cohort used by Zeng et al. [11]. We then present results, evaluate model goodness of fit, and describe predictions of metastasis‐free survival under two different treatment durations.

## 2 A Natural History Model of Breast Cancer

**FIGURE 1:**
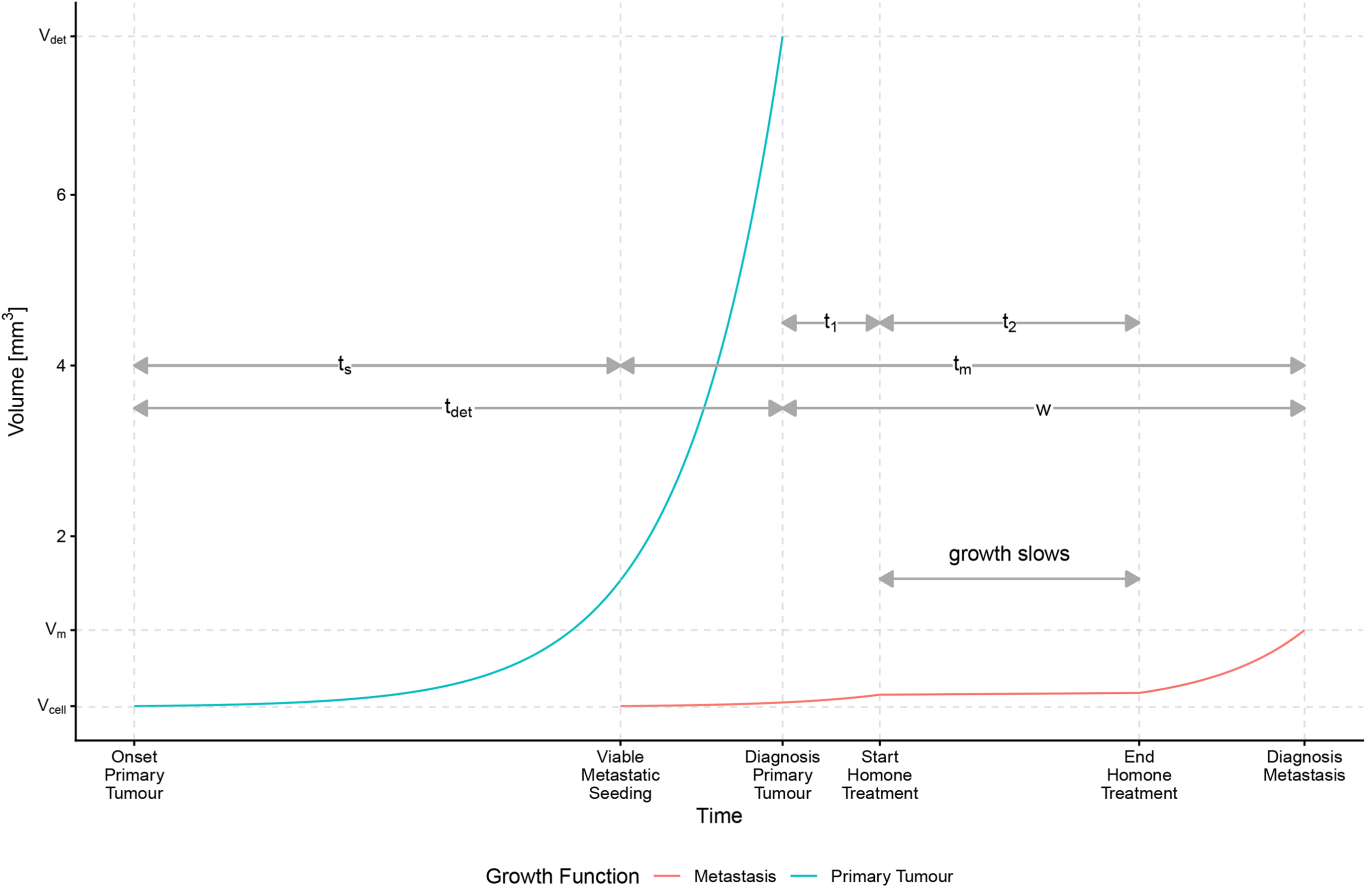
Representation of the breast cancer natural history of a hypothetical patient that had a viable seeding of metastasis before diagnosis of the primary tumour. The blue line represents the growth of the primary tumour, and the red one represents the metastatic growth. The scenario $\phi_{1}=\phi_{3}=1$ is represented, where there is no carryover effect and treatment is received only during the second time period, for a duration $t_{2}$, during which growth of the metastasis is slowed ($\phi_{2}>1$).

We develop a biologically inspired natural history model of breast cancer to study the effect of hormonal treatment on time from diagnosis of the primary tumour to detection of distant metastases, among patients with oestrogen receptor‐positive (ER+) breast cancer. In this model, a random effect on the (inverse) growth rate governs the variability across individuals in the rates of growth of primary tumours and the spread and growth of distant metastases. In the main data analysis of Section 4, we present a study in which all patients are subject to at most one period of treatment. After diagnosis, patients enter an initial treatment‐free phase. This is followed by a treatment period, typically lasting around five years. After the conclusion of treatment, patients are further monitored during an additional treatment‐free follow‐up period. Most patients are alive and have no detected distant metastases at the end of follow‐up, although, of course, some patients are diagnosed with distant metastases while still on treatment. Thus, with respect to being on treatment or not, in our main analysis, there are at most three distinct time periods. Although only one of the time periods includes treatment, in the main part of the article, we choose to describe our modelling approach for a slightly more general set‐up. This will clarify how our method can be easily adapted to other scenarios, such as those involving multiple treatments given at different times. We, in fact, explore an extension to four time periods in this paper, which we present as a sensitivity analysis in Appendix D. In describing the methodology in this section, we consider three distinct time periods, but include a parameter describing the effect of treatment on the growth of the distant metastasis in each of these periods. We allow the inverse growth rate of the distant metastasis to be modified by a (multiplicative) factor $\phi_{j}$ during the jth time period (with j=1,2,3). We describe below the assumptions that we make about the growth of the primary tumour, the process that generates distant metastases, and the effects of treatment. To aid the reader, we provide, in Table 1, a summary of the notation used in Sections 2 and 3. In addition, Figure 1 provides a visual representation of the modelling framework introduced in the following subsections. Specifically, it illustrates the special case $\phi_{1}=\phi_{3}=1$, which is the specification adopted in our main analysis (Section 4).

**TABLE 1:** Summary of the notation used.

Quantity	Description
**Random variables**	
*R*	random effect for the inverse growth rate
*V*_det_	random variable for tumour volume at symptomatic detection
*V*	random variable for tumour volume in the asymptomatic population at a particular time point
*W*	random variable for time from detection of the primary tumour to detection of distant metastases
*D*	denotes tumour diameter at the time of screening
*A*	denotes the presence of an asymptomatic tumour at a particular time point
*B*_0_	denotes the event of being screen‐detected
*B* ^c^	denotes the series of prior negative screens
*T*_s_	denotes the time from onset of breast tumour to first viable metastatic seeding
**Fixed quantities**	
*V*_m_ | *d*_m_	metastatic tumour volume | diameter (estimated)
*V*_0_ | *d*_0_	minimal tumour volume | diameter (fixed, d_0_ = 0.5 mm) to be detected at screening
*V*_cell_ | *d*_cell_	volume | diameter (fixed, d_cell = 0.01 mm) of a tumour cell
*t*_1_	time from diagnosis of the primary tumour to initiation of treatment (fixed per patient)
*t*_2_	treatment duration (fixed per patient)
*t*_m_	time from metastasis seeding to metastasis detection (fixed for a given *V*_*m*_, *R*, *t*_1_, *t*_1_)
**Model parameters**	
*τ*_1_ and *τ*_2_	shape and rate parameters of the gamma distribution of *R*
*φ* and *μ*	transformation of τ_1_ and τ_2_ to aid interpretability and improve identifiability
*η*	parameter of the hazard of symptomatic detection *h*_*V*_ *(t)*
*β*_1_ and *β*_2_	parameters of the logistic function that governs the sensitivity
*σ*	seeding rate of the non‐homogeneous Poisson process that governs the seeding process

### 2.1 Growth of the Primary Tumour

At the onset of the primary tumour, the first malignant cell has diameter $d_{\text{cell}}=0.01$ mm. We assume that the tumour is spherical and grows exponentially as

(1) $$\begin{array}{l} V(t,r)=V_{\text{cell}}\exp\left(\frac{t}{r}\right),\;t>0\;, \end{array}$$

where $V_{\text{cell}}$ is the volume corresponding to $d_{\text{cell}}$, t is time since onset, measured in years, and r is an inverse growth rate assumed to follow a Gamma distribution with scale parameter $\tau_{1}$ and rate $\tau_{2}$. V(t,r) represents tumour volume at time t for a tumour with inverse growth rate r. After onset, the primary tumour grows until it is diagnosed through the appearance of symptoms, or by screening, that is, by a mammographic image taken when there are no symptoms.

### 2.2 Modelling the Metastatic Seeding Process

From the moment of onset to diagnosis, a tumour has the possibility of shedding cells which subsequently seed in an organ far from the breast and give rise to distant metastases. We assume that until the primary tumour is detected, the process that generates viable distant metastatic seedings follows a non‐homogeneous Poisson process with an intensity function

(2) $$\begin{array}{l} \lambda (t,r)=\sigma^{*}D{\left(t,r\right)}^{k}D^{\prime }(t,r), \end{array}$$

where D(t,r) is the number of cell divisions that have been made by time t since onset of the primary tumour, $\sigma^{*}$ is a proportionality (scale) parameter governing the overall level of metastatic seeding, and k (k≥−1) is a parameter allowing for a power‐law relationship between the number of cell divisions and the rate of seeding, intended to capture the impact of genomic instability on the seeding rate. By viable metastatic seeding, we mean seeding events that result in deposits capable of sustained growth in the distant tissue and eventual clinical detection, since most seeded cells/deposits fail to progress [16]. For a detailed description of the choice of this intensity function, we refer the reader to Gasparini and Humphreys [17] (Section 2.2.1). We will later assume that λ(t,r)=0 for $t>t_{\det}$, where $t_{\det}$ represents the time from onset to diagnosis of the primary tumour. Ignoring this for the moment and assuming that tumour cells have an average volume of $V_{\text{cell}}$, the number of cell divisions, D(t,r), at time t, given an inverse growth rate r, can be calculated by solving

(3) $$\begin{array}{l} V_{\text{cell}}\times 2^{D(t,r)}=V(t,r)\Leftrightarrow D(t,r)=\frac{\log(V(t,r)/V_{\text{cell}})}{\log(2)}, \end{array}$$

where V(t,r) represents the volume at time t, of a tumour with an inverse growth rate r. The cumulative intensity function (from the intensity function of equation (2)) can be written as

(4) $$\begin{array}{l} \Lambda (t,r)=\int_{0}^{t}\lambda (u,r)du=\sigma {\left[\log\left(\frac{V(t,r)}{V_{\text{cell}}}\right)\right]}^{k+1}=\sigma {\left(\frac{t}{r}\right)}^{k+1}, \end{array}$$

where $\sigma =\sigma^{*}/[(k+1)\log{\left(2\right)}^{k+1}]$. Under the non‐homogenous Poisson process, the probability of having U=u viable seedings at time t (after onset of the primary tumour) is

(5) $$\begin{array}{l} P(U=u|T_{s}=t,R=r)=\frac{\Lambda {\left(t,r\right)}^{u}}{u!}\exp(-\Lambda (t,r)). \end{array}$$

We consider the random variable $T_{s}$, taking values $t_{s}$, to represent the time to first viable metastatic seeding. Its survival function is

(6) $$\begin{array}{ll} S\left(t_{s}|R=r\right) & =P\left(U=0|T_{s}=t_{s},R=r\right)\\ & =\exp\left(-\frac{\sigma }{r^{k+1}}t_{s}^{k+1}\right)\;,t_{s}\geq 0, \end{array}$$

its hazard function is

(7) $$\begin{array}{l} h\left(t_{s}|R=r\right)=\frac{\sigma }{r^{k+1}}(k+1)t_{s}^{k}\;,t\geq 0, \end{array}$$

and its probability density function is

(8) $$\begin{array}{l} f_{T_{s}}\left(t_{s}|R=r\right)=\frac{\sigma }{r^{k+1}}(k+1)t_{s}^{k}\exp\left(-\frac{\sigma }{r^{k+1}}t_{s}^{k+1}\right). \end{array}$$

As mentioned above, we will assume that no seeding takes place after the time of diagnosis of the primary tumour (as a result of surgery [18]). Consequently, the survival function, hazard function, and probability density function are defined only for $t_{s}\leq t_{\det}$, because $t_{s}$ is the time from onset to the first viable seeding event and $t_{\det}$ is the time from onset to detection of the primary tumour. With reference to Figure 1, seeding can occur only between “Onset Primary Tumour” and “Diagnosis Primary Tumour”.

### 2.3 Time to Detection of Distant Metastases and the Effect of Hormonal Treatment

If distant metastases occur before the detection of the primary tumour, we assume that, from the time of seeding, they grow exponentially, starting from the size of a single cell, with an inverse growth rate that is perfectly correlated with the one of the primary tumour (see Figure 1). As previously noted, variability in the (inverse) growth rates is captured by a gamma‐distributed random effect. For a patient with at least one viable metastatic seeding prior to the detection of the primary tumour, the detection of distant metastases is assumed to coincide with the time when the first seeded metastasis reaches a fixed volume $V_{m}$. We will estimate the value of $V_{m}$ by including it as a model parameter. Our approach therefore implicitly assumes that the first‐seeded metastasis will be the one which is detected first. Under the above conditions, Gasparini and Humphreys [17] derived the distribution of time to detection of the first seeded distant metastasis (counted from the time that the primary tumour was diagnosed), conditional on the size of the primary tumour, and fitted a model for this event time based on a sample of breast cancer patients. Despite modelling being based on a very simplified representation of the complex metastatic process, the model was shown to capture the relationship between primary tumour size and time to distant metastases well. In the current paper, we additionally incorporate an effect of treatment given in the years following diagnosis of the primary tumour. In our main analysis, we suppose that there are three distinct periods where the inverse growth rate is modified by a multiplicative factor ($\phi_{1}$, $\phi_{2}$, $\phi_{3}$, respectively). Additional time periods can easily be incorporated as we show later in additional analyses. In the case of three distinct time periods, the volume of the distant metastasis at time w (measured from the diagnosis of the primary tumour) will be

(9) $$\begin{array}{ll} & M(w,r)\\ & \;=\left\{\begin{array}{ll} V_{\text{cell}}\exp\left(\frac{t_{\det}-t_{s}}{r}\right)\; & \text{if}\;w=0,\\ V_{\text{cell}}\exp\left(\frac{t_{\det}-t_{s}}{r}+\frac{w}{\phi_{1}r}\right)\; & \text{if}\;0<w\leq t_{1},\\ V_{\text{cell}}\exp\left(\frac{t_{\det}-t_{s}}{r}+\frac{t_{1}}{\phi_{1}r}+\frac{w-t_{1}}{\phi_{2}r}\right)\; & \text{if}\;t_{1}<w\leq t_{1}+t_{2},\\ V_{\text{cell}}\exp\left(\frac{t_{\det}-t_{s}}{r}+\frac{t_{1}}{\phi_{1}r}\right.\; & \\ \left.+\frac{t_{2}}{\phi_{2}r}+\frac{w-t_{1}-t_{2}}{\phi_{3}r}\right)\; & \text{if}\;w>t_{1}+t_{2}, \end{array}\right.\; \end{array}$$

where $t_{1}$ is the length of the first time period and $t_{2}$ is the length of the second time period. Figure 1 depicts the interval from “Diagnosis Primary Tumour” to “Start Hormone Treatment” ($t_{1}$), during which no treatment effect is assumed ($\phi_{1}=1$); the interval from “Start Hormone Treatment” to “End Hormone Treatment” ($t_{2}$), during which the treatment effect is present ($\phi_{2}>1$); and the interval from “End Hormone Treatment” to “Diagnosis Metastasis” ($w-t_{1}-t_{2}$), during which no carryover effect is assumed ($\phi_{3}=1$). As already mentioned, as a sensitivity analysis, we later consider an extension to four time periods (and estimate two treatment effects) in order to incorporate a carryover effect of treatment.

#### 2.3.1 A Model for Time to Metastatic Detection

Using Equation (9), and solving for w when $M(w,r)=V_{m}$, we can write the time from diagnosis of the primary tumour to the metastatic diagnosis w as

(10) $$\begin{array}{l} w=\left\{\begin{array}{ll} \phi_{1}r\log\left(\frac{V_{m}}{V_{\text{cell}}\exp\left(\frac{t_{\det}-t_{s}}{r}\right)}\right)\; & \text{if}\;w\leq t_{1},\\ t_{1}+\phi_{2}r\log\left(\frac{V_{m}}{\begin{array}{c} V_{\text{cell}}\exp\left(\frac{t_{\det}-t_{s}}{r}\right)\\ +\frac{t_{1}}{\phi_{1}r} \end{array}}\right)\; & \text{if}\;t_{1}<w\leq t_{1}+t_{2},\\ t_{1}+t_{2}+\phi_{3}r\log\left(\frac{V_{m}}{\begin{array}{c} V_{\text{cell}}\exp\left(\frac{t_{\det}-t_{s}}{r}\right)\\ +\frac{t_{1}}{\phi_{1}r}+\frac{t_{2}}{\phi_{2}r} \end{array}}\right)\; & \text{if}\;w>t_{1}+t_{2}. \end{array}\right. \end{array}$$

It follows that the time from onset to first metastatic seeding is

(11) $$\begin{array}{l} t_{s}=\left\{\begin{array}{ll} \frac{w}{\phi_{1}}+r\log\left(\frac{V_{\det}}{V_{m}}\right)\; & \text{if}\;w\leq t_{1},\\ \frac{w}{\phi_{2}}+\frac{t_{1}}{\phi_{1}}-\frac{t_{1}}{\phi_{2}}+r\log\left(\frac{V_{\det}}{V_{m}}\right)\; & \text{if}\;t_{1}<w\leq t_{1}+t_{2},\\ \frac{w}{\phi_{3}}+\frac{t_{1}}{\phi_{1}}-\frac{t_{1}}{\phi_{3}}+\frac{t_{2}}{\phi_{2}}\; & \\ -\;\frac{t_{2}}{\phi_{3}}+r\log\left(\frac{V_{\det}}{V_{m}}\right)\; & \text{if}\;w>t_{1}+t_{2}. \end{array}\right. \end{array}$$

Applying the change‐of‐variable technique (to equation (8) with equation (11)) we can write the density of W as

(12) $$\begin{array}{ll} & f_{W}\left(w|R=r,V_{\det}=v\right)\\ & \;=\left\{\begin{array}{ll} \frac{\sigma (k+1)}{\phi_{1}r}{\left[\frac{w}{\phi_{1}r}+\log\left(\frac{v}{V_{m}}\right)\right]}^{k}\; & \\ \;\times \exp\left\{-\sigma {\left[\frac{w}{\phi_{1}r}+\log\left(\frac{v}{V_{m}}\right)\right]}^{k+1}\right\}\; & \text{if}\;w\leq t_{1},\\ \frac{\sigma (k+1)}{\phi_{2}r}{\left[\frac{w}{\phi_{2}r}+\frac{t_{1}}{\phi_{1}r}-\frac{t_{1}}{\phi_{2}r}+\log\left(\frac{v}{V_{m}}\right)\right]}^{k}\; & \\ \;\times \exp\left\{-\sigma \left[\frac{w}{\phi_{2}r}+\frac{t_{1}}{\phi_{1}r}-\frac{t_{1}}{\phi_{2}r}\right.\right.\; & \\ \;\left.{\left.+\log\left(\frac{v}{V_{m}}\right)\right]}^{k+1}\right\}\; & \text{if}\;t_{1}<w\leq t_{1}+t_{2},\\ \frac{\sigma (k+1)}{\phi_{3}r}\left[\frac{w}{\phi_{3}r}+\frac{t_{1}}{\phi_{1}r}-\frac{t_{1}}{\phi_{3}r}+\frac{t_{2}}{\phi_{2}r}\right.\; & \\ \;{\left.-\frac{t_{2}}{\phi_{3}r}+\log\left(\frac{v}{V_{m}}\right)\right]}^{k}\; & \\ \;\times \exp\left\{-\sigma \left[\frac{w}{\phi_{3}r}+\frac{t_{1}}{\phi_{1}r}-\frac{t_{1}}{\phi_{3}r}+\frac{t_{2}}{\phi_{2}r}\right.\right.\; & \\ \;\left.{\left.-\frac{t_{2}}{\phi_{3}r}+\log\left(\frac{v}{V_{m}}\right)\right]}^{k+1}\right\}\; & \text{if}\;w>t_{1}+t_{2}, \end{array}\right. \end{array}$$

and the hazard function of W as

(13) $$\begin{array}{ll} & h_{W}\left(w|R=r,V_{\det}=v\right)\\ & \;=\left\{\begin{array}{ll} \frac{\sigma }{\phi_{1}r}(k+1){\left[\frac{w}{\phi_{1}r}+\log\left(\frac{v}{V_{m}}\right)\right]}^{k}\; & \text{if}\;w\leq t_{1},\\ \frac{\sigma }{\phi_{2}r}(k+1)\left[\frac{w}{\phi_{2}r}+\frac{t_{1}}{\phi_{1}r}-\frac{t_{1}}{\phi_{2}r}\right.\; & \\ \;{\left.+\log\left(\frac{v}{V_{m}}\right)\right]}^{k}\; & \text{if}\;t_{1}<w\leq t_{1}+t_{2},\\ \frac{\sigma }{\phi_{3}r}(k+1)\left[\frac{w}{\phi_{3}r}+\frac{t_{1}}{\phi_{1}r}-\frac{t_{1}}{\phi_{3}r}+\frac{t_{2}}{\phi_{2}r}\right.\; & \\ {\left.\;-\frac{t_{2}}{\phi_{3}r}+\log\left(\frac{v}{V_{m}}\right)\right]}^{k}\; & \text{if}\;w>t_{1}+t_{2}. \end{array}\right. \end{array}$$

We now incorporate the earlier introduced assumption about removing metastatic seeding after diagnosis of the primary tumour. Imposing that the last possible time to have a viable seeding is at the time of diagnosis, constrains the hazard rate of detection of metastases to be zero for $w>t_{m}$. In the case of a viable seeding occurring at the exact time of primary tumour diagnosis, $t_{m}$ would be

(14) $$t_{m}=\left\{\begin{array}{ll} \phi_{1}r\log\left(\frac{V_{m}}{V_{\text{cell}}}\right)\; & \text{if}\;w<t_{1},\\ \phi_{2}r\log\left(\frac{V_{m}}{V_{\text{cell}}}\right)+t_{1}-\frac{\phi_{2}t_{1}}{\phi_{1}}\; & \text{if}\;t_{1}<w<t_{1}+t_{2},\\ \phi_{3}r\log\left(\frac{V_{m}}{V_{\text{cell}}}\right)\; & \\ +t_{1}+t_{2}-\frac{\phi_{3}t_{1}}{\phi_{1}}-\frac{\phi_{3}t_{2}}{\phi_{2}}\; & \text{if}\;w>t_{1}+t_{2}. \end{array}\right.$$

Hence, during the first time period (i.e., $w\leq t_{1}$) the survival function is

(15) $$\begin{array}{ll} & S_{W}\left(w|R=r,V_{\det}=v\right)\\ & =\;\left\{\begin{array}{ll} \exp\left\{-\sigma \left(\frac{w}{\phi_{1}r}\right.\right.\; & \\ \;\left.{\left.+\log\left(\frac{v}{V_{m}}\right)\right)}^{k+1}\right\}\; & \text{if}\;w\leq \phi_{1}r\log\left(\frac{V_{m}}{V_{\text{cell}}}\right),\\ \exp\left\{-\sigma {\left(\log\left(\frac{v}{V_{\text{cell}}}\right)\right)}^{k+1}\right\}\; & \text{if}\;w>\phi_{1}r\log\left(\frac{V_{m}}{V_{\text{cell}}}\right), \end{array}\right. \end{array}$$

where the thresholds for w follow from Equation (14). During the second time period (i.e., $t_{1}<w\leq t_{1}+t_{2}$) it is

(16) $$\begin{array}{ll} & S_{W}\left(w|R=r,V_{\det}=v\right)\\ & =\;\left\{\begin{array}{ll} \exp\left\{-\sigma \left(\frac{w}{\phi_{2}r}+\frac{t_{1}}{\phi_{1}r}-\frac{t_{1}}{\phi_{2}r}\right.\right.\; & \text{if}\;w\leq \phi_{2}r\log\left(\frac{V_{m}}{V_{\text{cell}}}\right)\\ \;\left.{\left.+\log\left(\frac{v}{V_{m}}\right)\right)}^{k+1}\right\}\; & \;+t_{1}-\frac{\phi_{2}t_{1}}{\phi_{1}},\\ \exp\left\{-\sigma {\left(\log\left(\frac{v}{V_{\text{cell}}}\right)\right)}^{k+1}\right\}\; & \text{if}\;w>\phi_{2}r\log\left(\frac{V_{m}}{V_{\text{cell}}}\right)\\ \; & \;+t_{1}-\frac{\phi_{2}t_{1}}{\phi_{1}}. \end{array}\right. \end{array}$$

and, during the third time period (i.e., $w>t_{1}+t_{2}$) it is

(17) $$\begin{array}{ll} & S_{W}\left(w|R=r,V_{\det}=v\right)\\ & =\;\left\{\begin{array}{ll} \exp\left\{-\sigma \left(\frac{w}{\phi_{3}r}+\frac{t_{1}}{\phi_{1}r}-\frac{t_{1}}{\phi_{3}r}\right.\right.\; & \\ \;+\frac{t_{2}}{\phi_{2}r}-\frac{t_{2}}{\phi_{3}r}\; & \text{if}\;w\leq \phi_{3}r\log\left(\frac{V_{m}}{V_{\text{cell}}}\right)\\ \;\left.{\left.+\log\left(\frac{v}{V_{m}}\right)\right)}^{k+1}\right\}\; & \;+t_{1}+t_{2}-\frac{\phi_{3}t_{1}}{\phi_{1}}-\frac{\phi_{3}t_{2}}{\phi_{2}},\\ \exp\left\{-\sigma {\left(\log\left(\frac{v}{V_{\text{cell}}}\right)\right)}^{k+1}\right\}\; & \text{if}\;w>\phi_{3}r\log\left(\frac{V_{m}}{V_{\text{cell}}}\right)\\ \; & \;+t_{1}+t_{2}-\frac{\phi_{3}t_{1}}{\phi_{1}}-\frac{\phi_{3}t_{2}}{\phi_{2}}. \end{array}\right. \end{array}$$

We note that our model is a statistical cure model for which the cure proportion—representing the probability of no seeded metastasis at the detection of the primary tumour—does not depend on the inverse growth rate or on the length of the treatment.

## 3 Likelihood Function

It is possible to write a likelihood for the joint density of size of primary tumour and time to event (i.e., distant metastasis detection), conditional on mode of detection (symptomatic or screen‐detected), timing of prior negative screens, and treatment regime, in terms of the model parameters. In writing this likelihood, we first write likelihood contributions for different types of events for a scenario in the absence of screening and then develop the procedure further to account for screening. Estimation of the model parameters relies on differences observed in tumour characteristics across individuals according to mode of detection and screening history. In this section, we outline the intuition behind the derivation of the likelihood function; the full derivation is provided in Appendix A.

### 3.1 A Cohort of Incident Cases of Breast Cancer

We describe how to estimate the natural history model described in Section 2 using a likelihood approach for a cohort of incident cases. In Section 4.1, we describe a cohort of invasive oestrogen receptor‐positive breast cancer cases which are followed prospectively for the detection of metastasis. To derive the likelihood, we work under a stable disease assumption, that is, that the onset of new tumours and the symptomatic detection process are constant across calendar time. This implies that, in the absence of screening, the distribution of tumour size at detection is stationary. Using this assumption, Isheden and Humphreys [19] showed two important results that we will use. First, they proved that, in the absence of screening, at any point in time, the probability density function of tumour sizes in the asymptomatic population is proportional to the probability density function of tumour size at detection divided by the hazard of becoming symptomatic

(18) $$\begin{array}{l} f_{V}(v|\text{having an asymptomatic tumour})\propto \frac{f_{V_{\det}}(v)}{h_{V}(v)}, \end{array}$$

where V is the random variable for tumour size at any time point, $V_{\det}$ is the tumour size at symptomatic detection, and $h_{V}(v)$ is the hazard of symptomatic detection of the primary tumour, which is a function of tumour size. If it is assumed that the minimum allowed volume for detection of the primary tumour is $V_{0}$ and that $h_{V}(v)=\eta v$ (for $v>V_{0}$), then, based on the exponential growth model with a gamma random effect for the inverse growth rate, it can be shown (see Plevritis et al. [20]) that

(19) $$\begin{array}{l} f_{V_{\det}}(v)=\eta \tau_{1}\frac{\tau_{2}^{\tau_{1}}}{{\left(\tau_{2}+\eta \left(v-V_{0}\right)\right)}^{\tau_{1}+1}},v>V_{0}. \end{array}$$

Second, Isheden and Humphreys [19] showed that the distribution of inverse growth rates among asymptomatic patients with a tumour of size v is the same as the distribution of inverse growth rates among symptomatically diagnosed patients with a diagnosed tumour of size v, specifically

(20) $$\begin{array}{ll} & f_{R}\left(r|\text{having an asymptomatic tumour of size}\;v\right)\\ & \;=f_{R}\left(r|V_{\det}=v\right) \end{array}$$

where under our modelling assumptions (see Plevritis et al. [20]), the conditional distribution takes the form

(21) $$\begin{array}{ll} f_{R}\left(r|V_{\det}=v\right) & =\frac{\left[\tau_{2}+\eta \left(v-V_{0}\right)\right]}{\Gamma \left(\tau_{1}+1\right)}{\left\{r\left[\tau_{2}+\eta \left(v-V_{0}\right)\right]\right\}}^{\left(\tau_{1}+1\right)-1}\\ & \;\exp\left\{-r\left[\tau_{2}+\eta \left(v-V_{0}\right)\right]\right\}. \end{array}$$

We remind the reader that a detailed description of the used random variables and parameters can be found in Table 1.

### 3.2 In the Absence of Screening

In Appendix A.1, we present the derivation of the likelihood contributions for breast cancer patients who never attended screening. Censoring and metastatic events can occur in different periods with respect to treatment, and patients' contributions change accordingly. A distant metastasis diagnosis can occur in any of the three time periods. In Appendix A.1, we describe the derivation of the likelihood contributions of (distant metastasis) events, left censored data, that is, from patients diagnosed with metastasis at the detection of their primary tumour, and right censored data, that is, from patients that did not experience an event during their follow‐up. In Appendix B, we describe a simulation study that we carried out to validate our derivation of the likelihood function. This simulation is based on a scenario in which screening is not offered to any member of the population. We note that when estimating the model without screening, we need to make a parameter restriction ($\tau_{1}=\tau_{2}$) for parameter identifiability purposes [20].

### 3.3 In the Presence of Screening

We extend our previous derivation of the likelihood to account for the presence of breast cancer screening. The complete derivation of the likelihood contributions for events, right and left censored with a history of screening attendance is presented in Appendix A.2. To validate this extended likelihood and our implementation of it, we conducted a simulation study, which is described in Appendix C.

In the Stockholm and Gotland health region of Sweden, where our study is based (see Section 4 for further details about the data), all women are invited to screening every two years from the age of 40, with the aim of detecting breast cancer early. An X‐ray is taken to the mammary gland and analysed by two independent radiologists to determine the presence of a tumour. If they find a suspicious mass, the woman is re‐called for further examinations. If the pathological analysis of the mass turns out to be malignant, the woman is considered diagnosed at screening. We model the sensitivity of this process with a logistic function

(22) $$\begin{array}{ll} & P(\text{tumour will be detected at screening}|D=d)\\ & \;=\frac{\exp\left(\beta_{0}+\beta_{1}d\right)}{1+\exp\left(\beta_{0}+\beta_{1}d\right)}, \end{array}$$

for $d>d_{0}$, where D is a random variable for tumour diameter at the time of screening and $d_{0}=0.5\text{mm}$ is the diameter corresponding to a spherical tumour of volume $V_{0}$. To calculate the probability of being screen‐detected at each possible screening, we use the back‐calculation algorithm described in Isheden and Humphreys [19].

The likelihood contribution of each individual is calculated on the joint distribution of tumour size at detection and time to metastasis, conditional on screening history and mode of detection. Using the notation of Table 1 the likelihood contribution for screen‐detected patients is

(23) $$\begin{array}{l} L_{v,w}\propto P\left(B_{0}|V=v\right)P(V=v,W=w|A)P\left(B^{c}|A,V=v,W=w\right). \end{array}$$

while for symptomatically detected patients it is

(24) $$\begin{array}{l} L_{v,w}\propto P\left(W=w,V_{\det}=v\right)P\left(B^{c}|V_{\det}=v,W=w\right). \end{array}$$

The derivations of these contributions are based on the stable disease assumptions and the two theorems of Isheden and Humphreys [19] (equations (18) and (20) herein), and can be found in Appendix A.2.

## 4 Analysis of Data From a Cohort of ER+ Breast Cancer Patients

### 4.1 Cohort Description

We analyse data from a cohort of invasive breast cancer patients diagnosed in the Stockholm‐Gotland health region of Sweden between July 2005 and August 2020. Data from the Stockholm‐Gotland Quality Register for Breast Cancer was merged with data from the Stockholm‐Gotland mammography screening register and data from the Swedish Prescribed Drug Register [21]. We used the first source for the information on tumour size at detection and date of metastatic recurrence, the second source for the information on mode of detection and screening history (i.e., dates of all previously attended screens), and the third source for the information on length of adjuvant hormonal treatment. Treatment discontinuation was recorded when there was no collection of either drug (i.e., tamoxifen or aromatase inhibitor) for four consecutive months. Treatment duration was defined as the period from the collection of the first package of tablets to three months after the last collection, as each prescription covers a three‐month supply. The primary event of interest was the diagnosis of metastasis, with survival time measured as metastasis‐free survival (MFS) from the date of primary tumour diagnosis. For patients with missing information on the date of metastasis but who died from breast cancer (28 patients), we imputed the date of death as the metastasis diagnosis date. We did this because these few patients most likely had symptoms of advanced disease very close to death and were not formally staged for distant metastasis. Patients were censored at the time of contralateral breast cancer diagnosis, emigration, death due to other causes, or the end of follow‐up on August 31, 2020. Only 139 patients developed contralateral breast cancer, of whom 131 received hormonal treatment, with a median treatment duration of 3.24 years. For additional details regarding the registers and data retrieval process, we refer the reader to Zeng et al. [11].

The dataset we worked with was selected from an original sample of 17,578 ER+ patients (ER ≥10% in the analysed tissue). We selected regular screeners who were screened and symptomatically detected according to the definition of Holm et al. [22]. After further removing patients with missing data on tumour size, mode of detection, or those who experienced local recurrence (as this factor violates the assumption of ceasing metastatic seeding at diagnosis), we were left with 9,716 patients, among which there were 299 distant metastatic events during follow‐up. For these 299 patients, the median time to metastasis was 3.91 years (mean = 4.49, IQR = [2.36–6.15], maximum = 13.76). Characteristics of the final sample are summarised in Table 2.

**TABLE 2:** Characteristics of ER+ breast cancer patients included in the Swedish study of hormonal treatment and metastatic events. Numbers (with percentages) or total number of years, or, for tumour diameter, median (and lower and upper quartiles) are provided.

	Detection mode	
	Screen	Symptomatic
Full cohort		
Number of patients	7,150 (73.6%)	2,566 (26.4%)
Negative screens prior to diagnosis		
No Screens	807 (11.3%)	1 (0.0%)
1 screen	659 (9.2%)	319 (12.4%)
2 screens	705 (9.9%)	351 (13.7%)
3 or more screens	4,979 (69.6%)	1,895 (73.9%)
Tumour diameter (mm)	14 (10, 20)	17 (12, 25)
Metastatic recurrence	162 (2.27%)	137 (8.75%)
Total time at risk (years)	47,340.80	16,378.54
Total time on treatment (years)	25,876.61	9,486.62

**FIGURE 2:**
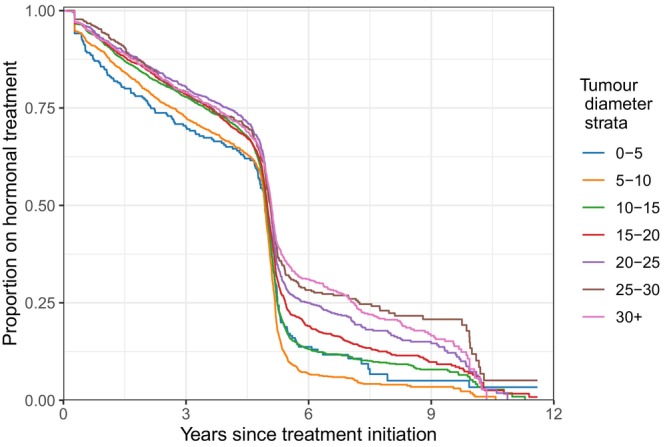
Estimates of the proportion of patients on hormonal treatment by time from the start of treatment, for different categories of primary tumour diameters (Kaplan‐Meier plots). Treatment discontinuation is defined as no drug collection for four consecutive months. A patient is treated as censored when she has a metastatic event, reaches the end of follow‐up (August 31, 2020), experiences a contralateral breast cancer, a second primary breast cancer, dies, or emigrates.

In the Swedish dataset, we observed a clear positive association between treatment duration and tumour size (Figure 2). We note that 29 patients did not initiate treatment; of these, 18 were diagnosed with distant metastases, including 3 cases where distant metastases were already present at the time of primary tumour diagnosis. These patients are not included in Figure 2.

### 4.2 Model Estimation

Using the likelihood described in Section 3, we fitted our model to the Swedish data described in Section 4.1. We fitted the model by maximum likelihood; computational details are provided in Appendix A.3. Table 3 displays the estimated values of the model parameters (with their 95% confidence interval). We fixed $\phi_{1}=\phi_{3}=1$ in our analysis, which is consistent with the study's context, and k=4 as in Gasparini and Humphreys [17]. Re‐parameterising the gamma‐distributed inverse growth rate R we obtain $\mu =\frac{\tau_{1}}{\tau_{2}}$ and $\psi =\frac{1}{\tau_{1}}$, where μ represents the expected value of the inverse growth rate. We estimated a mean doubling time of 293.173 and a median doubling time of 248.197. These estimates are in line with estimates of growth rates of ER+ tumours obtained using serial images [23, 24, 25, 26].

**TABLE 3:** Parameter Estimates with 95% Confidence Intervals computed with non‐parametric bootstrap (1500 samples), based on the Swedish study of hormonal treatment and metastatic events. The unit of measurement for $d_{m}$ is the millimetre.

Parameter	Estimate	95% CI
Growth rate		
μ	1.158	(1.026,1.331)
ψ	0.476	(0.395,0.618)
Symptomatic detection		
−logη	9.792	(9.485,10.033)
Screening sensitivity		
$\beta_{1}$	−4.713	(−4.849,−4.602)
$\beta_{2}$	0.426	(0.401,0.451)
Rate of seeding		
logσ	−17.681	(−17.860,−17.512)
Treatment effect		
$\phi_{2}$	2.414	(1.698,3.098)
$d_{m}$	0.907	(0.686,1.443)

η represents the hazard parameter associated with symptomatic detection, while $\beta_{1}$ and $\beta_{2}$ are parameters defining screening sensitivity (with these parameter values the screening sensitivities for tumours of 5, 10, and 15 mm are respectively 0.070, 0.389, and 0.843). The parameter $\phi_{2}$ captures the treatment effect; a value of 2.414 indicates that the rate of growth of a seeded metastasis is estimated to be slowed more than two‐fold. One should, however, be cautious in interpreting this biologically since its value is strongly correlated to $d_{m}$, which itself is unknown and anyway represents a vast simplification of the metastases detection process. The (fixed) diameter at which metastases are deemed to be detected, $d_{m}$, was not estimated in the study by Gasparini and Humphreys [17]; instead, it was fixed at 0.5 mm. In this study, its estimated value was larger.

### 4.3 Goodness of Fit

**FIGURE 3:**
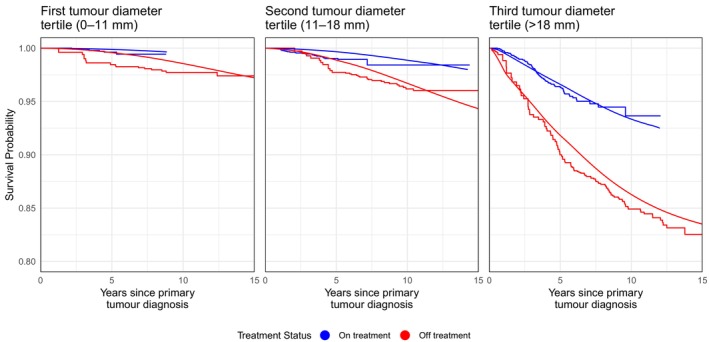
Predicted and observed distant metastatic event survival for patients treated (blue line) and non‐treated (red line). The treatment curve is truncated at the time the last person in the treatment group stops treatment or is censored. The off‐treatment curve is truncated at 15 years as no events occur beyond this point.

To evaluate the goodness of fit of the estimated model, we compare the expected survival (time from diagnosis to metastatic recurrence) with the observed survival of individuals treated and not treated with endocrine therapy. Depicting survival is challenging since patients switch between the treated and non‐treated groups at different times. To plot the observed survival, we use the extended Kaplan‐Meier estimator described by Snapinn et al. [27]. Although, under most conditions, survival comparisons based on this estimator do not have a causal interpretation [28], it provides a useful means for us to assess how our model fits the data. At an event time, this extended Kaplan‐Meier estimator is calculated as $S_{k}(t)=\prod_{j:t_{j}\leq t}\{1-d_{jk}/n_{jk}\}$, where $n_{jk}$ is the number of people at risk in group k (the groups are treated and non‐treated in our case) at time $t_{j}$, and $d_{jk}$ is the number of individuals with an event at time $t_{j}$ in group k. We stratify our analysis by tertiles of tumour size. The Kaplan‐Meier plots in Figure 3 include all patients except those with distant metastases already at the time of diagnosis of the primary tumour.

In order to describe how well our fitted model aligns with the observed survival patterns, we use a procedure based on model‐based predictions that mimics the extended Kaplan‐Meier estimator. We divide time into short intervals of length dt (in practice, we used dt=0.1) and at the start of each interval, we determine the current at‐risk sets (either on or off treatment). Then, for each patient, we evaluate the model‐predicted conditional probability of surviving to time t+dt, given survival up to time t, tumour size, and timing of negative screens prior to diagnosis. For this calculation, we use the procedure described in Section 2.5 of Gasparini and Humphreys [17]. For each treatment group, these probabilities are then averaged over all patients. Their sequential products can then be plotted—these appear as smooth functions alongside the Kaplan‐Meier plots in Figure 3.

### 4.4 Model‐Based Prediction

**FIGURE 4:**
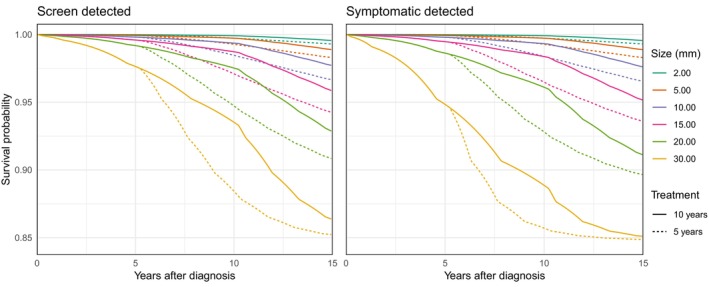
Predicted distant metastatic event survival functions by tumour size and treatment duration. The plot on the left‐hand side depicts the predicted survival functions for screen‐detected patients with three previous negative screens taken every two years. The plot on the right‐hand side shows the predicted survival functions for symptomatically detected patients with three previous negative screens every two years and a diagnosis one year after their last negative screen. The solid lines represent survival for patients assigned to treatment for 10 years, whilst the dashed lines represent survival for patients assigned to treatment for 5 years.

Based on survival functions described in Sections 2 and 3 and the parameter estimates presented in Table 3, we can calculate expected survival distributions under hypothetical scenarios in which patients receive hormonal treatment for different lengths of time. We compare 5‐year and 10‐year treatment durations (from diagnosis of the primary tumour) for particular types of patients in terms of mode of detection and tumour size. We assume that each patient attended screening at regular intervals of two years, and that symptomatic cases attended their most recent negative screen exactly one year before diagnosis. We calculate survival functions, conditional on no distant metastases at detection of the primary tumour, for 5‐year and 10‐year treatment durations for six different tumour sizes at detection. These are plotted as dashed and solid lines, respectively, in Figure 4. The survival functions are identical during the first five years. For each tumour size, the area between the solid and dashed lines quantifies the survival gain from extending treatment from 5 to 10 years. We note that according to our estimates, the 10‐year metastasis‐free survival would improve from 92.8% to 96.1% for a symptomatic patient with a 20 mm tumour with ten years (instead of five years) of hormonal treatment.

### 4.5 Carryover Effect of Treatment and Additional Sensitivity Analysis

In Appendix D, we present stratified analyses by several factors that can potentially modify the effect of hormonal treatment (lymph node status, grade and chemotherapy). We also performed analyses for different lengths of follow‐up, as a means of investigating differences in treatment effect according to time since treatment initiation. Neither across different strata, nor across different lengths of follow‐up did we observe substantial differences in treatment effect. However, in stratified analyses, there were notable differences in the estimated rates of seeding, with higher rates observed for more aggressive tumour types. This is not altogether surprising and reflects a need for extending the model to incorporate the factors on which we stratified, but in a more biologically motivated fashion. We come back to this point in the Discussion. Parameter estimates for the stratified analyses are presented in Appendix D.

Furthermore, we investigated how incorporating a carryover effect of treatment (i.e., a residual effect on metastatic growth after treatment cessation) in our model would influence our results, since prior studies have suggested that hormonal therapy may have a carryover effect persisting for years after treatment cessation [1, 2, 29]. In practice, it is unclear how a carryover effect could manifest itself. As a pragmatic approach, and as a sensitivity analysis, we extended our natural history model to allow treatment to influence metastasis growth for an additional post‐treatment period, assumed to be equal in length to the time on treatment (e.g., 3 years of tamoxifen treatment would be followed by a 3‐year carryover). During this carryover window, we allow for the metastasis growth to remain slowed via an extra effect parameter, after which the effect ceases for the remaining follow‐up time. This is, of course, ad hoc and serves only as a sensitivity analysis. The methodological extension and the results of this analysis are reported in Appendix D. Essentially, we allow for four distinct time periods and estimate a $\phi_{2}$ and $\phi_{3}$, while fixing $\phi_{1}=\phi_{4}=1$. Under this specification, estimating the carryover parameter did not materially alter other parameter estimates relative to those obtained in the non‐carryover model (our main analysis), but a likelihood ratio test provided evidence in favour of a carryover effect, albeit one which is reasonably modest in magnitude. Only small differences in the expected survival curves of the two models were observed. Incorporating the carryover effect did not substantially attenuate the survival benefit associated with extending treatment from 5 to 10 years (see Figure S1 in the Supporting Information).

## 5 Discussion

We have described a biologically motivated natural history model of breast cancer for modelling the time‐dependent effect of hormonal treatment on metastasis‐free survival. It models the joint probability of tumour size at detection and metastasis‐free survival time conditional on the mode of detection (i.e., screening or symptoms) and screening history. We fitted our model to data from a cohort of oestrogen receptor‐positive patients (ER+) diagnosed with invasive breast cancer between 2007 and 2020 in the Stockholm‐Gotland region. A major purpose of our work has been to gain insight into the potential effect of modifying adjuvant treatment duration in ER+ breast cancer patients.

Tamoxifen acts as a selective oestrogen receptor modulator (SERM) by binding to oestrogen receptors in breast tissue and thereby inhibiting oestrogen‐driven tumour cell proliferation (it has a cytostatic effect, leaving the cells in the G0 or G1 phase [30]). Aromatase inhibitors act upstream by suppressing oestrogen synthesis, so reducing circulating oestrogen levels [5]. Because ER‐positive tumours depend on oestrogen signalling for growth, both therapies are expected to attenuate tumour progression. Endocrine therapies are generally regarded as predominantly cytostatic rather than directly cytotoxic [31]. Although oestrogen deprivation and related agents can activate apoptotic pathways in preclinical models, clinical eradication of established metastatic disease is uncommon [32, 33]. We therefore modelled the effect of hormonal therapy as slowing metastatic growth rather than as killing metastatic cells.

We note that a post‐treatment (carryover) effect, which we have explored in sensitivity analyses, has been suggested by several researchers and supported empirically. To address this, in sensitivity analyses, we incorporated a carryover component by assuming that the effect persists for a duration equal to the treatment period. We estimated a statistically significant carryover effect; however, its impact on 15‐year survival was small relative to the model with fixed $\phi_{3}=1$ (Figure S1). We note that our aim has not been to study in detail the nature of a carryover effect. Further analyses might, for example, allow $\phi_{3}$ to decay with time rather than assuming a constant effect over a fixed period.

Other works have tried to characterise the effect of hormonal treatment on patient outcomes. Using standard regression methods, Zeng et al. [11] compared disease‐free survival (conditional on being disease‐free 5 years after diagnosis) between patients stopping hormonal treatment at 5 years and patients with extended treatment duration and after adjusting for tumour characteristics, demonstrated that extending hormonal treatment beyond five years was associated with improved disease‐free survival (HR: 0.72 [0.55‐0.95]). Accurately quantifying the effect of altering treatment duration on survival (from the time of diagnosis) is challenging without conducting a large and expensive clinical trial. Additionally, clinical trials often have stringent inclusion criteria, limiting their generalizability to the broader population. In contrast, our study leverages population‐level data, providing insights into the real‐world effectiveness of treatment across a more diverse cohort. The modelling approach presented here offers a tool to explore the potential impact of modifying treatment duration, serving as a cost‐effective alternative for hypothesis generation and decision support.

Several mathematical approaches for modelling tumour growth and treatment have been described and used in cancer biology research, and these have been used in so‐called in‐silico models and are often based on experimental data. Enderling and Chaplain [34] provide examples of such models, which are based on partial differential equations. Yin et al. [35] review diverse modelling strategies for tumour dynamics and treatment effects. Malinzi et al. [36] and Watanabe et al. [37] illustrate how different therapies can be modelled mathematically. None of these are directly relevant to the type of data we have in our study, though, where we make use of large population‐based registry data, including data on screening and time‐dependent treatment data. We note that a more general approach for evaluating the contributions of treatment and screening in reducing mortality, for population registry data, is described by Berry et al. [38]. This uses data on survival and treatment but does not explicitly model the time‐dependent effect of treatment and does not explicitly connect the treatment/patient outcomes to the natural history of the cancer. Instead, it models outcomes conditional on tumour and patient characteristics at the diagnosis of the primary tumour.

Using our model, we studied the potential impact of different treatment durations on metastasis‐free survival. This can, of course, only be considered as a causal comparison under the conditions that our model is correct and that there are no unmeasured confounders. As well as being clear that this is intended as an approximation of the impact of altering treatment duration, it is important to acknowledge that our model assumes the effect of treatment ($\phi_{2}$) to be equal for all patients, which in practice is unlikely to be true. If it is not true, then the estimated effect will, however, represent, at least approximately, an average effect, even if the treatment duration is not independent of the treatment effect, which would be the case if, for example, side effects (leading to withdrawal) were associated with the efficacy of the treatment.

As mentioned in the Introduction, it would of course be possible to apply more common statistical approaches that are used for analysing time‐varying treatments and time‐to‐event outcomes. For instance, time‐dependent flexible parametric survival models could be used to capture the complex relationships between tumour volume and a cure proportion. We briefly explored the use of these models with our data, however, the implementations of this model, and even the more standard Cox proportional hazards model with time varying exposures, are, as far as we are aware, all based on a memoryless counting process, and due to this, it is not possible, within available software, to construct predictions that would be necessary for assessing goodness of fit via the use of for example, extended Kaplan‐Meier plots. On the other hand, joint modelling approaches [14, 39], which model the time‐varying treatment jointly with the time‐to‐event outcome, are potentially a more promising approach for the nature of the data analyses in the current paper, that is, incorporating prediction based on a time‐varying treatment. Several techniques for joint modelling of longitudinal binary markers and time‐to‐event outcomes have been described, and different approaches to modelling the hazard as a function of treatment can be specified. For example, the hazard can be described as a function of treatment duration, and lag effects can be incorporated [40, 41]. Another possibility would be estimating the causal effect of time‐varying treatment utilising (i) marginal structural models with inverse probability weighting of treatment [15, 42], (ii) g‐formula [43], or (iii) doubly robust methods like targeted maximum likelihood estimation (TMLE) [44, 45, 46]. Some of these methods have been developed for advanced scenarios where treatment regimes depend on time‐dependent covariates (e.g., for HIV‐related treatments [47]).

Whilst all of the above approaches are broadly useful, our method is more specific and captures key characteristics of the metastatic process and treatment effect and their interplay, which are grounded on biologically‐reasonable assumptions and not incorporated in/captured by existing implementations of the above approaches. In particular, our approach estimates:

- a relationship between tumour volume and cure fraction;
- a complex (biologically motivated) relationship between the interplay of timing of treatment, tumour growth rate and volume of the primary tumour (at diagnosis), and the hazard rate of detection of distant metastases in the non‐cured sub‐population (see equation (13)).

Despite difficulties of comparisons with common approaches, we plan in a future project to compare the performance of the natural history model and some of the approaches mentioned above. This, however, is beyond the scope of the current article.

Our approach is also based on other strong assumptions. For instance, tamoxifen and aromatase inhibitors are considered as a single hormonal treatment, despite the possibility of differing effects between the two. It would be interesting to explore separate treatment effects for tamoxifen and aromatase inhibitors. Additionally, the model assumes a common volume for detecting metastases, which may oversimplify the underlying biological variability. The tumour volume is also assumed to be spherical, with the diameter measured at its largest part, potentially overestimating the actual size. Our model assumes exponential growth, and in principle, other growth functions could be considered. Given the nature of our data, we are also only able to model the net growth of tumours. The use of a fixed multiplicative treatment effect across the population might fail to capture the heterogeneity in treatment responses among individuals. The model assumes that the inverse growth rates of the primary tumour and metastases are perfectly correlated, whereas, in reality, this correlation is likely to be lower. It may be possible to model the correlation between the inverse growth rates of the primary tumour and distant metastases, although care would need to be taken with parameter identifiability. Furthermore, the dormancy period that metastases might encounter is not characterised by any sub‐processes of the model.

One omission in our work is that we have not accounted for the apoptotic effect of chemotherapy on seeded metastases. We have essentially ignored this source of heterogeneity in metastasis‐free survival. This somewhat compromises the interpretation of our model for metastases seeding. However, parameters $d_{m}$ and $\phi_{2}$, when interpreted together rather than individually, provide meaningful information on the time to metastasis and ensure a good fit to the data, as shown in Figure S1. In this model, the cure proportion (i.e., the proportion of patients that will never be diagnosed with metastasis) and the rate at which metastases are diagnosed are dependent only on tumour size at diagnosis (and hormonal treatment). An approach to address this would be to model additional tumour characteristics such as the number of lymph nodes affected at the time of surgery, as a function of growth rate (i.e., not just tumour size) and to incorporate an effect of chemotherapy on the cure proportion. The process of lymph node spread has already been integrated into the tumour progression model, without considering treatment, by Gasparini and Humphreys [48], and this might be able to provide a solid foundation for such an adjustment. Ideally, one should model all tumour characteristics associated with metastasis‐free survival, as they influence the decision of the oncologist to prescribe chemotherapy. Moreover, tumour grade may have an effect on spread [49], so the model could be extended to accommodate this as well. To provide an initial assessment of how chemotherapy may influence metastatic seeding and interact with the effect of hormonal therapy, we conducted stratified analyses by lymph‐node status, tumour grade, and chemotherapy use, as reported in the Results section and in Appendix D.

Despite all of the above‐mentioned assumptions (and other assumptions implicit in our modelling), we believe that our approach provides an interesting and useful tool for approximating the time‐dependent effect of hormonal treatment. Our approach could be useful to analyse data from, for example, a trial to study the effect of different doses of tamoxifen on metastasis‐free survival. Our aim has been to explore the use of (causal) modelling based on biological assumptions as a tool for studying time‐dependent treatment effects in (breast) cancer, which is otherwise difficult with standard statistical approaches. While our approach has limitations, as outlined in this Discussion, we believe it can contribute to a better understanding of cancer progression and treatment effects.

## Funding

This work was supported by Vetenskapsrådet (Grant No. 2023‐02063) and Cancerfonden (Grant No. 2023‐2686).

## Disclosure

A.G. is an employee of Red Door Analytics AB, which had no role in the design, conduct, or reporting of this research, and did not influence the interpretation of the findings or the decision to publish. All statements in this report, including its findings and conclusions, are solely those of the authors and do not necessarily represent the views of Red Door Analytics AB.

## Conflicts of Interest

The authors declare no conflicts of interest.

## Supporting information




**Data S1.** Supporting Information.

## Data Availability

The data that support the findings of this study are available from the corresponding author upon request after ethical approvals have been obtained from the Swedish Ethical Review Board.
