# Supplementary material for: Time‐Varying Hormonal Treatment and Metastasis‐Free Survival Among ER+ Breast Cancer Patients: A Natural History Modelling Approach
Source: Stat Med. 2026 Apr 2;45(8-9):e70504. doi: 10.1002/sim.70504 (PMC13044503; doi:10.1002/sim.70504)
Supplement: Supplementary file 1 — Data S1. Supporting Information. [file SIM-45-0-s001.pdf]

APPENDIX

A LIKELIHOOD CONTRIBUTIONS

In this section of the appendix, we provide a detailed derivation of the likelihood contributions for the natural history model introduced in Section 2. We first derive the likelihood for patients who did not attend screening, specifying the contributions for events, left-censored, and right-censored observations according to whether the event or censoring occurred before, during, or after treatment (see Figure 1). Then, we proceed to describe how screening changes the likelihood both for screen and symptomatically detected cancer with a history of screening attendance.

A.1 In the absence of screening

We describe here how to derive the likelihood for the joint distribution of the time to metastasis and the tumour volume at detection in an unscreened population, where detection occurs only through symptoms.

A.1.1 Contributions of events

To derive the joint probability for the time from diagnosis to metastasis (W) and tumour volume at detection (V_{det}), we multiply the conditional probability of W given V_{det}, by the probability of V_{det}. Using the law of total probability, we calculate $f_{W|V_{\text{det}}}(w|v)$ by marginalising $f_{W|V_{\text{det}},R}(w|v,r)$ (see equation (12)) over the gamma-distributed inverse growth rate (R). When marginalising, the lower limit of the integral over the support of R (ξ) corresponds to the smallest/fastest possible inverse growth rate capable of generating a metastasis that is later detected at time w (see Figure 1for a graphical intuition of w). Since the seeding process is assumed to cease upon diagnosis of the primary tumour, this lower limit reflects the scenario where the metastasis was seeded precisely at the time of primary tumour diagnosis. We can write the joint probability density function as

$$\begin{aligned} f_{W,V_{\text{det}}}(w,v) &= f_{W|V_{\text{det}}}(w \mid v)\, f_{V_{\text{det}}}(v) \\ &= f_{V_{\text{det}}}(v) \int_{\xi}^{+\infty} f_{W|R,V_{\text{det}}}\big(w \mid r, v\big)\, f_{R|V_{\text{det}}}(r \mid v)\,\mathrm{d}r. \end{aligned} \tag{A1}$$

where

$$\xi = \begin{cases} \dfrac{w}{\phi_1 \log\left(\dfrac{V_m}{V_{cell}}\right)} & \text{if } w < t_1, \\ \dfrac{w - t_1 + \dfrac{\phi_2 t_1}{\phi_1}}{\phi_2 \log\left(\dfrac{V_m}{V_{cell}}\right)} & \text{if } t_1 \le w < t_1 + t_2, \\ \dfrac{w - t_1 - t_2 + \dfrac{\phi_3 t_1}{\phi_1} + \dfrac{\phi_3 t_2}{\phi_2}}{\phi_3 \log\left(\dfrac{V_m}{V_{cell}}\right)} & \text{if } w \ge t_1 + t_2. \end{cases} \tag{A2}$$

We can then write the likelihood contributions for events, for the three time periods as:

- Events during the first time period

$$P(W = w, V_{det} = v) = \eta\tau_1 \frac{\tau_2^{\tau_1}}{(\tau_2 + \eta(v - V_0))^{\tau_1+1}} \int_{\xi}^{+\infty} f_W(w|R, V_{det}) f_R(r|V_{det}) dr, \tag{A3}$$

where

$$f_W(w|R = r, V_{det} = v) = \frac{\sigma}{\phi_1 r}(k+1)\left[\frac{w}{\phi_1 r} + \log\left(\frac{v}{V_m}\right)\right]^k \exp\left\{-\sigma\left[\frac{w}{\phi_1 r} + \log\left(\frac{v}{V_m}\right)\right]^{k+1}\right\}, \tag{A4}$$

$f_R(r|V_{det})$ can be found in equation (21), and

$$\xi = \frac{w}{\phi_1 \log(V_m/V_{cell})}; \tag{A5}$$

- Events during the second time period

$$P(W = w, V_{det} = v) = \eta\tau_1 \frac{\tau_2^{\tau_1}}{(\tau_2 + \eta(v - V_0))^{\tau_1+1}} \int_{\xi}^{+\infty} f_W(w|R, V_{det}) f_R(r|V_{det}) dr, \tag{A6}$$

where

$$f_W(w|R = r, V_{det} = v) = \frac{\sigma}{\phi_2 r}(k+1)\left[\frac{w}{\phi_2 r} + \frac{t_1}{\phi_1} - \frac{t_1}{\phi_2} + \log\left(\frac{v}{V_m}\right)\right]^k \times \exp\left\{-\sigma\left[\frac{w}{\phi_2 r} + \frac{t_1}{\phi_1} - \frac{t_1}{\phi_2} + \log\left(\frac{v}{V_m}\right)\right]^{k+1}\right\}, \tag{A7}$$

and

$$\xi = \frac{w - t_1 + \phi_2 t_1/\phi_1}{\phi_2 \log(V_m/V_{cell})}; \tag{A8}$$

- Events during the third time period

$$P(W = w, V_{\text{det}} = v) = \eta\tau_1 \frac{\tau_2^{\tau_1}}{(\tau_2 + \eta(v - V_0))^{\tau_1+1}} \int_{\xi}^{+\infty} f_W(w|R, V_{\text{det}}) f_R(r|V_{\text{det}}) dr, \tag{A9}$$

where

$$f_W(w|V_{\text{det}} = v, R = r) = \frac{\sigma}{\phi_3 r}(k+1)\left(\frac{w}{\phi_3 r} + \frac{t_1}{\phi_1 r} - \frac{t_1}{\phi_3 r} + \frac{t_2}{\phi_2 r} - \frac{t_2}{\phi_3 r} + \log\frac{v}{V_{\text{m}}}\right)^k \times$$
$$\exp\left[-\sigma\left(\frac{w}{\phi_3 r} + \frac{t_1}{\phi_1 r} - \frac{t_1}{\phi_3 r} + \frac{t_2}{\phi_2 r} - \frac{t_2}{\phi_3 r} + \log\frac{v}{V_{\text{m}}}\right)^{k+1}\right], \tag{A10}$$

and

$$\xi = \frac{w - t_1 - t_2 + \phi_3 t_1/\phi_1 + \phi_3 t_2/\phi_2}{\phi_3 \log(V_{\text{m}}/V_{\text{cell}})}. \tag{A11}$$

A.1.2 Left censored contributions

Left-censored patients present with metastases at diagnosis, implying that metastatic lesions reached the detection threshold d_{m} before the primary breast cancer was diagnosed. Consequently, their time to metastasis (W) is negative. From equation (15), the probability of having developed metastases by the time of diagnosis, conditional on V_{det} and R, is

$$F(W \leq 0 \mid V_{\text{det}} = v, R) = 1 - \exp\left[-\sigma\left(\log\frac{v}{V_{\text{m}}}\right)^{k+1}\right], \tag{A12}$$

which does not depend on R. Therefore, the likelihood contribution for a left-censored patient can be written as

$$P(W \leq 0, V_{\text{det}} = v) = f_{V_{\text{det}}}(v)\left\{1 - \exp\left[-\sigma\left(\log\frac{v}{V_{\text{m}}}\right)^{k+1}\right]\right\}. \tag{A13}$$

A.1.3 Right censored contributions

For patients without distant metastases at the end of follow-up, the likelihood contribution depends on the time period in which censoring occurs. The general form for the contribution of right-censored patients is

$$P(W > w, V_{\text{det}} = v) = f_{V_{\text{det}}}(v) \int_R S_W(w|R, V_{\text{det}}) f_R(r|V_{\text{det}} = v) dr$$
$$= f_{V_{\text{det}}}(v)\left[\int_0^{\xi} S_W(w|R, V_{\text{det}}) f_R(r|V_{\text{det}} = v) dr + \int_{\xi}^{\infty} S_W(w|R, V_{\text{det}}) f_R(r|V_{\text{det}} = v) dr\right]. \tag{A14}$$

As was also the case for the events, there are three scenarios:

- right censored during the first time period ($w < t_1$)

$$P(W > w, V_{\mathrm{det}} = v) = f_{V_{\mathrm{det}}}(v) \left\{ \exp\left\{ -\sigma \left[\log\left(\frac{v}{V_{\mathrm{cell}}}\right) \right]^{k+1} \right\} F_R(\xi | V_{\mathrm{det}} = v) + \int_{\xi}^{\infty} \exp\left[-\sigma \left(\frac{w}{\phi_1 r} + \log\left(\frac{v}{V_{\mathrm{m}}}\right) \right)^{k+1} \right] f_R(r | V_{\mathrm{det}} = v) dr \right\}, \tag{A15}$$

where

$$\xi = \frac{w}{\phi_1 \log(V_{\mathrm{m}}/V_{\mathrm{cell}})}; \tag{A16}$$

- right censored the second time period ($t_1 < w \leq t_1 + t_2$)

$$P(W > w, V_{\mathrm{det}} = v) = f_{V_{\mathrm{det}}}(v) \left\{ \exp\left\{ -\sigma \left[\log\left(\frac{v}{V_{\mathrm{cell}}}\right) \right]^{k+1} \right\} F_R(\xi | V_{\mathrm{det}} = v) + \int_{\xi}^{\infty} \exp\left[-\sigma \left(\frac{w}{\phi_2 r} + \frac{t_1}{\phi_1 r} - \frac{t_1}{\phi_2 r} + \log \frac{v}{V_{\mathrm{m}}} \right)^{k+1} \right] f_R(r | V_{\mathrm{det}} = v) dr \right\}, \tag{A17}$$

where

$$\xi = \frac{w - t_1 + \phi_2 t_1/\phi_1}{\phi_2 \log(V_{\mathrm{m}}/V_{\mathrm{cell}})}; \tag{A18}$$

- right censored during the third time period ($w > t_1 + t_2$)

$$P(W > w, V_{\mathrm{det}} = v) = f_{V_{\mathrm{det}}}(v) \left\{ \exp\left\{ -\sigma \left[\log\left(\frac{v}{V_{\mathrm{cell}}}\right) \right]^{k+1} \right\} F_R(\xi | V_{\mathrm{det}} = v) + \int_{\xi}^{\infty} \exp\left[-\sigma (\frac{w}{\phi_3 r} + \frac{t_1}{\phi_1 r} - \frac{t_1}{\phi_3 r} + \frac{t_2}{\phi_2 r} - \frac{t_2}{\phi_3 r} + \log \frac{v}{V_{\mathrm{m}}})^{k+1} \right] f_R(r | V_{\mathrm{det}} = v) dr \right\}, \tag{A19}$$

where

$$\xi = \frac{w - t_1 - t_2 + \phi_3 t_1/\phi_1 + \phi_3 t_2/\phi_2}{\phi_3 \log(V_{\mathrm{m}}/V_{\mathrm{cell}})}. \tag{A20}$$

Under our modelling assumptions, the conditional cumulative density function of the inverse growth rate is

$$F_R(r | V_{\mathrm{det}} = v) = \frac{\gamma(\tau_1 + 1, r[\tau_2 + \eta(v - V_{\mathrm{cell}})])}{\Gamma(\tau_1 + 1)},$$

where $\Gamma(.)$ is the lower incomplete gamma function.

A.2 In the presence of screening

As described in Section 3.3, in Sweden women aged 45 years and older are invited to biennial screening up to age 75. In the following subsections, we derive the likelihood contributions for screen-detected and symptom-detected patients with a history of screening attendance.

A.2.1 Screen detected cancers

For screen-detected patients with a history of negative screens, written in terms of probabilities, the likelihood can be written as

$$L \propto P(B_0|V = v)P(V = v, W = w|A)P(B^c|A, V = v, W = w) \tag{A21}$$

where V is a random variable for volume at screen detection, and W is time to distant metastasis since primary tumour diagnosis (see Table 1). For individuals with no previous history of screening attendance the term $P(B^c|A, V, W)$ can be omitted.

Making use of the theoretical results of Isheden and Humphreys[19] that were described in Section 3.1 (equations (18) and (20)), Isheden and Humphreys[17] showed how to write this likelihood in terms of the parameters of the submodels for tumour growth, metastatic spread, symptomatic detection and screen detection. The likelihood contributions they derived are the same as the ones described below but adapted to treatment following diagnosis of the primary tumour.

Screen detected cancers with metastatic events

We can write the likelihood contribution for screen-detected patients with a metastatic event as

$$L_{v,w} \propto P(B_0|V = v) \times \frac{f_{V_{\text{det}}}(v)}{h_v(v)} \times \int_{\xi}^{+\infty} \left[\prod_{q=1}^{p} P(B_q^c|R = r, V = v) \right] f_W(w|R = r, V = v) f_R(r|V_{\text{det}} = v) dr, \tag{A22}$$

where, according to which time period the event occurs, $f_W(w|R = r, V = v)$ is substituted with the corresponding term of equation (12), and ξ is substituted with the corresponding term of equation (A2), exactly as in the case in the absence of screening of Appendix A.1.

Left censored contributions

For screen-detected patients who had distant metastases already at the time of diagnosis of the primary tumour, the likelihood contribution can be written as

$$L_{v,w} \propto P(B_0|V = v) \times \frac{f_{V_{\text{det}}}(v)}{h_V(v)} \times \left\{ 1 - \exp\left[-\sigma \left(\log \frac{v}{V_{\text{m}}} \right)^{k+1} \right] \right\} \times \int_{0}^{+\infty} \left[\prod_{q=1}^{p} P(B_q^c|R = r, V = v) \right] f_R(r|V_{\text{det}} = v) dr. \tag{A23}$$

Right censored contributions

The likelihood contribution of individuals that have not had a metastasis by the end of follow-up can be written as

$$L_{v,w} \propto P(B_0|V=v) \times \frac{f_{V_{\text{det}}}(v)}{h_V(v)} \times \left\{ \int_{\xi}^{+\infty} \left[\prod_{q=1}^{p} P(B_q^c|R=r, V=v) \right] S_W(w|V=v, R=r) \right.$$
$$\left. \times f_R(r|V_{\text{det}}=v)dr + \int_{0}^{\xi} \left[\prod_{q=1}^{p} P(B_q^c|R=r, V=v) \right] \exp\left\{ -\sigma \left[\log\left(\frac{v}{V_{\text{cell}}} \right) \right]^{k+1} \right\} f_R(r|V_{\text{det}}=v)dr \right\} \quad \text{(A24)}$$

where $S_W(w|V=v, R=r)$ and ξ must be substituted with equation (15) and the first term of equation(A2) if the patient was censored during the first time period, with equation (16) and the second term of equation (A2) if the patient was censored during the second time period, and with equation (17) and the third term of equation (A2) if censored during the third time period.

A.2.2 Symptomatically detected cancers

The likelihood contribution for cases detected symptomatically and with a history of prior negative screens is

$$L_{v,w} \propto P(W=w, V_{\text{det}}=v)P(B^c|V_{\text{det}}=v, W=w). \quad \text{(A25)}$$

The equations for the contributions of events, left-censored, and right-censored observations are derived analogously to the equation for screen-detected cases in Section A.2.1. The final expressions correspond to equations (A22), (A23), (A24) but with the term $\frac{P(B_0|V=v)}{h_V(v)}$ excluded. For women with no prior (negative) screens who were symptomatically detected, the likelihood contribution is calculated using the equations provided in Appendix A.1.

A.3 Computational aspects

The likelihood was implemented with R language and optimised using `R` version 4.3.2[50]. For optimisation, we utilised the Hooke-Jeeves algorithm[51], a derivative-free method available in the R package `dfoptim`[52]. This algorithm, which is well-suited for optimising non-linear functions, did not encounter any convergence issues with our likelihood.

All the integrals presented in Appendix A.1 and A.2 have no closed form, so they must be computed numerically. For computational reasons, we approximate the probability density function of V_{det} using a probability mass function $f_{V_{\text{det}}} \approx P(V_{\text{det}} \in \mathcal{I}_i)$ where $\mathcal{I}$ is a set of volume intervals which under an assumption of spherical tumours correspond to diameter intervals (in millimetres) of $\{[0.5, 1.5), [1.5, 2.5), [2.5, 7.5), [7.5, 12.5), \dots, [67.5, 72.5), [72.5, 85), [85, 95), \dots, [145, 155)\}$. To compute the probability of each interval, we use its mid-point. This approximation has also been used both in Isheden and Humphreys[19], and Gasparini and Humphreys[17].

The term $P(B_q^c|R=r, V=v)$ is computed using the backward projection algorithm described in Weedon-Fekjær et al.[53]. Essentially, it calculates the probability of a cancer not being detected at the most recent negative screen, given the tumour size

at detection, by summing over all possible sizes up to and including the detected size. This procedure is then repeated for all q previously attended (negative) screens. For our likelihood model, we considered only the most recent three negative screens attended by each woman before diagnosis of their primary tumour.

B SIMULATION UNDER NO SCREENING

We simulated a single sample of 100,000 patients in the absence of screening from the set of parameters specified in Table 3 to check our specification of the likelihood and our implementation. Note that a single repetition with a large sample size is a valid approach to test the implementation of a method[54]. With no screening, the model is not fully identifiable, thus we fix $\mu = 1$ and $k = 4$ (this is similar to a parameter restriction used in Gasparini and Humphreys[17]) and estimate the rest of the parameters maximising the likelihood of the model. We set the detection diameter of the secondary tumour to $d_{\mathrm{m}} = 0.5$ mm, and the minimum detection diameter of the primary tumour at $d_0 = 0.5$ mm. The time from symptomatic diagnosis to start of the treatment is 2.5 years, and the treatment time is 8 years for everyone. We ended follow-up at 60 years after diagnosis, which is of course not realistic, but served well for checking the algorithm and ensuring a large number of events. All parameters were estimated without detectable bias (less than 1% on a relative scale).

Parameter	True values	Estimated	Profile Likelihood	P-value
ψ	0.5	0.505	672821.2	0.157
ϕ_2	1.5	1.497	672820.3	1.000
$-\log(\eta)$	9.1	9.098	672820.3	1.000
$\log\sigma$	-15.019	-15.014	672820.7	0.371
- log-Likelihood value		67, 2820.3		

TABLE S1 Results from a simulation conducted to verify the specification of the likelihood in the absence of screening. The *True values* column represents the parameter set used to generate a dataset of 50,000 patients. The profile likelihood value is obtained by optimising the likelihood while fixing each parameter, one at a time, to its true value. The final column presents the p-value from a likelihood ratio test.

C SIMULATION WITH SCREENING

We generated data using the same data-generation mechanism of Appendix B. The time to treatment initiation was set to 2.5 years and the treatment time to 8.5 years, with a maximum of 60 years of follow-up. We simulated a screening process with a 2-year interval since birth. The results of this simulation in terms of estimates of the model parameters are shown in Table S2 .

We observed that all differences between the likelihood of the full model and the profile likelihood values were small (within the 99% profile likelihood confidence intervals), except for β_1 and β_2. Nonetheless, the difference between these estimated

Parameter	True values	Estimated	Profile Likelihood	P-value
μ	0.55	0.541	41804.84	0.138
ψ	0.4	0.414	41804.49	0.221
β_1	-5	-4.383	41862.23	< 0.001
β_2	0.7	0.623	41825.70	< 0.001
ϕ_2	1.3	1.305	41806.00	0.034
$-\log \eta$	9.1	9.034	41805.44	0.065
$\log \sigma$	-15.019	-15.020	41803.74	1.000
- log-Likelihood value		$41,803.74$		

TABLE S2 Results from a simulation carried out to check specification of the likelihood in the presence of screening. The column *true values* corresponds to the set of parameters used to simulate a dataset of 10000 in-silico patients. The profile likelihood value is the likelihood value optimised while fixing each parameter (in turn) to their true value. The last column corresponds to a p-value based on a likelihood ratio test.

parameter values and the screening sensitivity function is not problematic, as the two sensitivity curves are closely aligned. In fact, Gasparini and Humphreys [17] have reported the same issue: this discrepancy arises because the probability of detecting small tumours during screening is extremely low, leading to insufficient cases of small tumours being detected.

D SENSITIVITY ANALYSIS

D.1 Stratified analysis

Here we report analyses of the effect of hormonal treatment, by different lengths of follow-up time (Table S3), and additional sensitivity analyses stratified by lymph node status (Table S4), tumour grade (Table S5), and chemotherapy status (Table S6). For all models, d_{m} was fixed to the value reported in Table 3 to enable comparison of ϕ_2 across model specifications. Table S3 presents parameter estimates obtained by censoring follow-up at 5 years and 10 years, to assess whether the treatment effect differs across shorter time windows. The results suggest that the estimated treatment effect increases with longer follow-up, although the estimates remain broadly comparable across follow-up windows.

Table S4 reports estimates from analyses stratified by lymph node status (node-positive vs. node-negative). Lymph node involvement is a well-established marker of more aggressive disease, and the model correspondingly estimates a higher metastasis rate and faster tumour growth on average among node-positive patients. Nevertheless, estimated treatment effects are similar in node-positive and node-negative groups and even reasonably similar across tumour grades, which represent disease severity in terms of the degree of cellular differentiation (there is, in any case, no clear dosage effect) (Table S5). Because tumour grade and lymph-node status strongly influence the clinical decision to administer chemotherapy, the estimated effect of hormonal treatment stratified by chemotherapy (Table S6) largely reflects patterns associated with these prognostic factors. Instead, to study chemotherapy effect, which, unlike hormonal therapy, is assumed to act through an apoptotic (cytotoxic) effect on tumour cells, it would be more relevant to investigate its impact on the cured proportion, i.e., the fraction of patients who

will never develop metastases. However, because grade and lymph-node status also drive treatment allocation, they constitute important confounders of the chemotherapy effect. The analysis could therefore adjust for these factors, for example, by using a copula-based approach to model the dependence between treatment assignment and prognosis. See the Discussion for additional comments on this topic.

D.2 Carryover effect

Several studies[1,2,29] have suggested the presence of a carryover effect of hormonal treatment that persists after treatment cessation. Our main analysis in this paper incorporates an effect of treatment that is not purely instantaneous in terms of an association with the rate of metastatic disease diagnosis, and this has been biologically motivated. This also varies according to tumour characteristics. As a sensitivity analysis we however here adapt our main model to incorporate an additional carryover effect. It is difficult to know exactly how a carryover effect would manifest itself. For our sensitivity analysis, we assume that its duration matches the duration of treatment. For example, if a woman received tamoxifen for 3 years, we assume the carryover effect would continue for an additional 3 years after stopping treatment. To incorporate this carryover effect, we introduced an additional time period t_3 with an associated parameter ϕ_3 to our natural history model described in Section 2. During t_3, the model is allowed to estimate a post-treatment effect (ϕ_3) under which metastasis growth is slowed. After t_3 ends, we assume no further effect of having been on treatment: from that point until the end of follow-up, the effect parameter is fixed to $\phi_4 = 1$. Using the same approach to that used for our main analysis, and similarly to equations (12) and (17), we obtain the probability density function and the associated survival function for this extension as

$$f_W(w \mid R = r, V_{\text{det}} = v) = \begin{cases} \dfrac{\sigma(k+1)}{\phi_3 r}\Big[\dfrac{w}{\phi_3 r} + \dfrac{t_1}{\phi_1 r} - \dfrac{t_1}{\phi_3 r} + \dfrac{t_2}{\phi_2 r} - \dfrac{t_2}{\phi_3 r} + \log\Big(\dfrac{v}{V_{\text{m}}}\Big)\Big]^k & \\ \quad \times \exp\Big\{-\sigma\Big[\dfrac{w}{\phi_3 r} + \dfrac{t_1}{\phi_1 r} - \dfrac{t_1}{\phi_3 r} + \dfrac{t_2}{\phi_2 r} - \dfrac{t_2}{\phi_3 r} + \log\Big(\dfrac{v}{V_{\text{m}}}\Big)\Big]^{k+1}\Big\} & \text{if } t_1 + t_2 < w \le t_1 + t_2 + t_3, \\ \dfrac{\sigma(k+1)}{\phi_4 r}\Big[\dfrac{w}{\phi_4 r} + \dfrac{t_1}{\phi_1 r} - \dfrac{t_1}{\phi_4 r} + \dfrac{t_2}{\phi_2 r} - \dfrac{t_2}{\phi_4 r} + \dfrac{t_3}{\phi_3 r} - \dfrac{t_3}{\phi_4 r} + \log\Big(\dfrac{v}{V_{\text{m}}}\Big)\Big]^k & \\ \quad \times \exp\Big\{-\sigma\Big[\dfrac{w}{\phi_4 r} + \dfrac{t_1}{\phi_1 r} - \dfrac{t_1}{\phi_4 r} + \dfrac{t_2}{\phi_2 r} - \dfrac{t_2}{\phi_4 r} + \dfrac{t_3}{\phi_3 r} - \dfrac{t_3}{\phi_4 r} + \log\Big(\dfrac{v}{V_{\text{m}}}\Big)\Big]^{k+1}\Big\} & \text{if } w > t_1 + t_2 + t_3, \end{cases} \tag{D26}$$

and

$$S_W(w \mid R = r, V_{\text{det}} = v) = \begin{cases} \exp\Big\{-\sigma\Big(\dfrac{w}{\phi_4 r} + \dfrac{t_1}{\phi_1 r} - \dfrac{t_1}{\phi_4 r}\dfrac{t_2}{\phi_2 r} - \dfrac{t_2}{\phi_4 r} + & \text{if } w \le \phi_4 r \log\Big(\dfrac{V_{\text{m}}}{V_{\text{cell}}}\Big) + t_1 + t_2 + t_3 - \dfrac{\phi_4 t_1}{\phi_1} - \dfrac{\phi_4 t_2}{\phi_2} - \dfrac{\phi_4 t_3}{\phi_3}, \\ \quad \dfrac{t_3}{\phi_3 r} - \dfrac{t_3}{\phi_4 r} + \log\Big(\dfrac{v}{V_{\text{m}}}\Big)\Big)^{k+1}\Big\} & \\ \exp\Big\{-\sigma\Big(\log\Big(\dfrac{v}{V_{\text{cell}}}\Big)\Big)^{k+1}\Big\} & \text{if } w > \phi_4 r \log\Big(\dfrac{V_{\text{m}}}{V_{\text{cell}}}\Big) + t_1 + t_2 + t_3 - \dfrac{\phi_4 t_1}{\phi_1} - \dfrac{\phi_4 t_2}{\phi_2} - \dfrac{\phi_4 t_3}{\phi_3}. \end{cases} \tag{D27}$$

Table S7 reports estimates from fitting the model, which incorporates a carryover period effect. Under this specification, all parameter estimates from the model with ϕ_3 fall within the confidence intervals of the ϕ_3-fixed model. Nevertheless, a likelihood ratio test comparing the two models indicates an improved fit to the data.

Figure S1 compares the ϕ_3-fixed and ϕ_3-estimated models in terms of predictions of survival probabilities (time to distant metastasis). The left panel shows 15-year survival for screen-detected cases with a screening history of two screens two years apart, contrasting 10-year treatment (solid line) with 5-year treatment (dashed line). The right panel shows the corresponding results for symptomatic cases detected one year after their last screen, with the same prior screening history. Observed survival is estimated using an extended Kaplan-Meier approach that accounts for the time-varying nature of treatment status (see Section 4.3), since individuals can move between the untreated and treated groups over follow-up. Expected survival is obtained by averaging, at each time point, the model-based survival probabilities over all individuals who are treated and untreated at that time point.

	Follow-up		
Parameter	Full (n events = 299)	10-year (n events = 287)	5-year (n events = 188)
μ	1.159 (1.026, 1.331)	1.128	1.054
ϕ	0.477 (0.395, 0.618)	0.458	0.444
$-\log\eta$	9.792 (9.485, 10.033)	9.807	9.743
$\log\sigma$	-17.681 (-17.860, -17.512)	-17.649	-17.660
β_1	-4.713 (-4.849, -4.602)	-4.704	-4.715
β_2	0.426 (0.401, 0.451)	0.431	0.444
ϕ_2	2.414 (1.698, 3.098)	2.382	2.098
d_m	0.907 (0.686, 1.443)	0.907	0.907

TABLE S3 Parameter estimates with d_m fixed for full (15.16 years), 10-year, and 5-year follow-up periods.

	Lymph Node	
Parameter	Positive (n = 2675)	Negative (n =6973)
μ	0.963	1.158
ϕ	0.463	0.516
$-\log\eta$	10.391	9.055
$\log\sigma$	-17.276	-18.239
β_1	-5.325	-4.859
β_2	0.380	0.465
ϕ_2	2.134	2.331
d_m	0.907	0.907

TABLE S4 Results of analyses stratified by lymph node status. Parameter estimates are obtained with d_m fixed at its value estimated in the main analysis.

Parameter	Grade		
	Grade 1 (n =2241)	Grade 2 (n =5420)	Grade 3 (n =1929)
μ	1.618	1.183	0.922
ϕ	1.345	0.434	0.749
$-\log\eta$	7.738	10.198	8.798
$\log\sigma$	-19.549	-17.675	-17.435
β_1	-5.913	-4.865	-5.246
β_2	0.543	0.426	0.331
ϕ_2	2.332	2.646	1.906
d_m	0.907	0.907	0.907

TABLE S5 Results of analyses stratified by tumour grade. Parameter estimates are obtained with d_m fixed at its value estimated in the main analysis.

Parameter	Chemotherapy	
	Non-treated (n =5708)	Treated (n =3976)
μ	1.151	1.201
ϕ	0.436	0.525
$-\log\eta$	8.890	10.332
$\log\sigma$	-18.230	-17.303
β_1	-4.905	-5.343
β_2	0.513	0.355
ϕ_2	2.714	2.131
d_m	0.907	0.907

TABLE S6 Results of analyses stratified by chemotherapy status. Parameter estimates are obtained with d_m fixed at its value estimated in the main analysis.

Parameter	ϕ_3	
	Fixed	Estimated
μ	1.159 (1.026, 1.331)	1.105
ϕ	0.477 (0.395, 0.618)	0.470
$\log\sigma$	-17.681 (-17.860, -17.512)	-17.596
$-\log\eta$	9.792 (9.485, 10.033)	9.739
β_1	-4.713 (-4.849, -4.602)	-4.726
β_2	0.426 (0.401, 0.451)	0.435
ϕ_2	2.414 (1.698, 3.098)	2.646
ϕ_3	1 (fixed)	1.416
ϕ_4	1 (fixed)	1 (fixed)
d_m	0.907 (0.686, 1.443)	0.907 (fixed)
$-\log l(\theta)$	22,321.54	22,316.04

TABLE S7 Parameter estimates for models with ϕ_3 fixed versus estimated, under the assumption that the post-treatment carryover effect of hormonal therapy persists for the same duration as the time on treatment. For the ϕ_3-estimated model, d_m was fixed to facilitate comparison of ϕ_2.

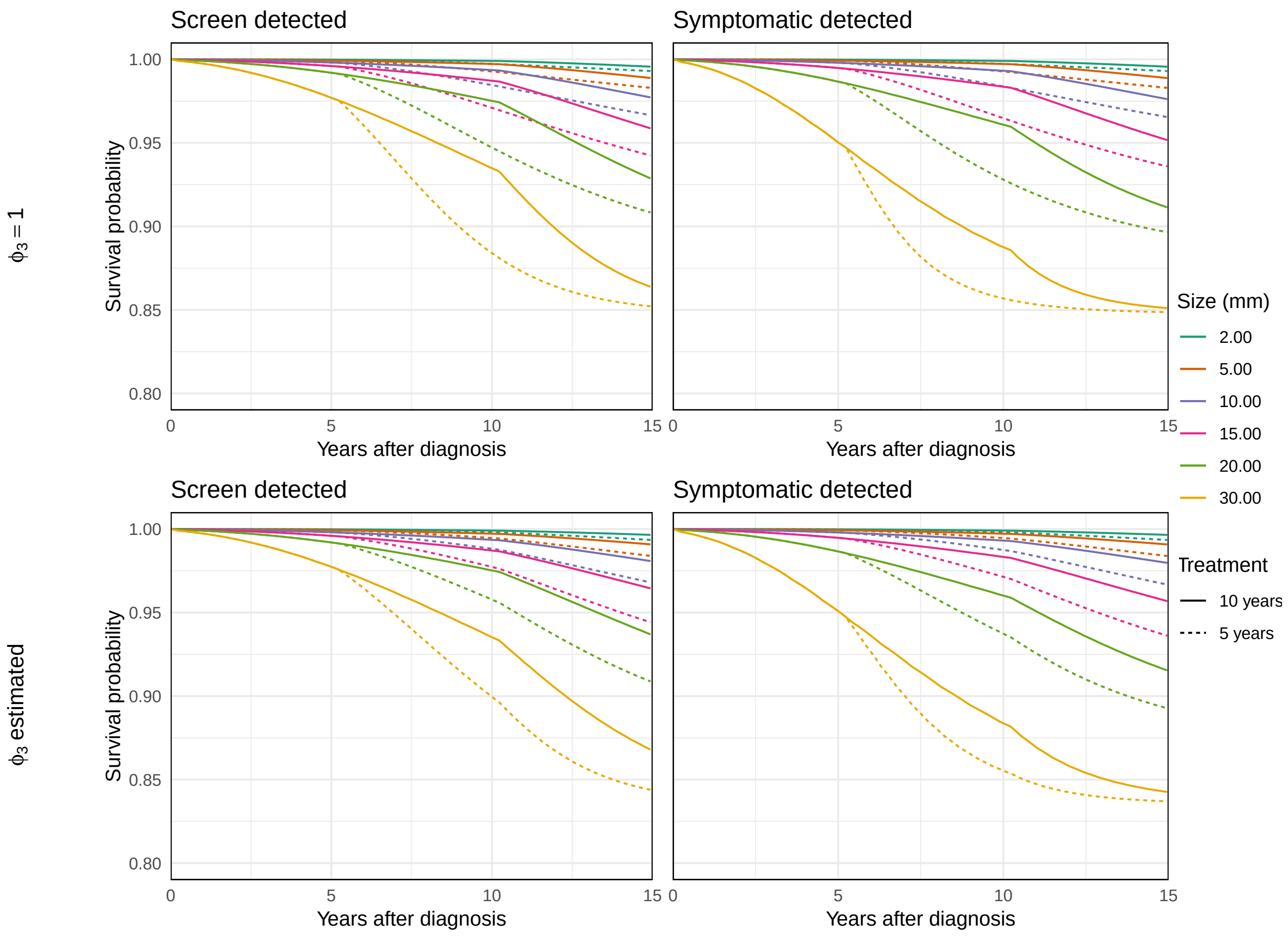

FIGURE S1 Predicted distant metastatic event survival functions by tumour size. Survival functions are shown using the parameter estimates in Table S7 , comparing 5-year treatment (dashed line) with 10-year treatment (solid line). The first row corresponds to the model with ϕ_3 fixed (i.e., no carryover effect). The second row corresponds to the model in which ϕ_3 is estimated, allowing for a post-treatment carryover effect whose duration is assumed to match the treatment duration.

REFERENCES

50. R Core Team. R: A Language and Environment for Statistical Computing. R Foundation for Statistical Computing, Vienna, Austria, 2023.
51. Kelley CT. Iterative Methods for Optimization. SIAM, 1999.
52. Varadhan R, Borchers HW, Bechard V. dfoptim: Derivative-Free Optimization. R package version 2023.1.0, 2023.
53. Weedon-Fekjær H, Tretli S, Aalen OO. Estimating Screening Test Sensitivity and Tumour Progression Using Tumour Size and Time since Previous Screening. *Statistical Methods in Medical Research* 2010; 19(5): 507–527.
54. Morris TP, White IR, Crowther MJ. Using Simulation Studies to Evaluate Statistical Methods. *Statistics in Medicine* 2019; 38(11): 2074–2102. doi: 10.1002/sim.8086.
